# Exploring selection signatures in the divergence and evolution of lipid droplet (LD) associated genes in major oilseed crops

**DOI:** 10.1186/s12864-024-10527-4

**Published:** 2024-07-01

**Authors:** Ramya Parakkunnel, Bhojaraja Naik K, Girimalla Vanishree, Anjitha George, Sripathy KV, Aruna YR, Udaya Bhaskar K, A Anandan, Sanjay Kumar

**Affiliations:** 1 ICAR- Indian Institute of Seed Science, Regional Station, GKVK Campus, Bengaluru, 560065 Karnataka India; 2 ICAR- Indian Institute of Seed Science, Mau, 275103 Uttar Pradesh India

**Keywords:** Germination, Lipid droplet, Lipoxygenase, Phospholipase D, Natural selection, Oleosin, Rancidity, TAG lipase

## Abstract

**Background:**

Oil bodies or lipid droplets (LDs) in the cytosol are the subcellular storage compartments of seeds and the sites of lipid metabolism providing energy to the germinating seeds. Major LD-associated proteins are lipoxygenases, phospholipaseD, oleosins, TAG-lipases, steroleosins, caleosins and SEIPINs; involved in facilitating germination and enhancing peroxidation resulting in off-flavours. However, how natural selection is balancing contradictory processes in lipid-rich seeds remains evasive. The present study was aimed at the prediction of selection signatures among orthologous clades in major oilseeds and the correlation of selection effect with gene expression.

**Results:**

The LD-associated genes from the major oil-bearing crops were analyzed to predict natural selection signatures in phylogenetically close-knit ortholog clusters to understand adaptive evolution. Positive selection was the major force driving the evolution and diversification of orthologs in a lineage-specific manner. Significant positive selection effects were found in 94 genes particularly in oleosin and TAG-lipases, purifying with excess of non-synonymous substitution in 44 genes while 35 genes were neutral to selection effects. No significant selection impact was noticed in Brassicaceae as against LOX genes of oil palm. A heavy load of deleterious mutations affecting selection signatures was detected in T-lineage oleosins and LOX genes of *Arachis hypogaea*. The T-lineage oleosin genes were involved in mainly anther, tapetum and anther wall morphogenesis. In *Ricinus communis* and *Sesamum indicum* > 85% of PLD genes were under selection whereas selection pressures were low in *Brassica juncea* and *Helianthus annuus*. Steroleosin, caleosin and SEIPINs with large roles in lipid droplet organization expressed mostly in seeds and were under considerable positive selection pressures. Expression divergence was evident among paralogs and homeologs with one gene attaining functional superiority compared to the other. The LOX gene *Glyma.13g347500* associated with off-flavor was not expressed during germination, rather its paralog *Glyma.13g347600* showed expression *in Glycine max*. PLD-α genes were expressed on all the tissues except the seed,δ genes in seed and meristem while β and γ genes expressed in the leaf.

**Conclusions:**

The genes involved in seed germination and lipid metabolism were under strong positive selection, although species differences were discernable. The present study identifies suitable candidate genes enhancing seed oil content and germination wherein directional selection can become more fruitful.

**Supplementary Information:**

The online version contains supplementary material available at 10.1186/s12864-024-10527-4.

## Background

Plant-based oils are important for food and industrial uses with major players being sunflower, olive, castor, sesame, soybean, peanut, corn, coconut, cottonseed, rice bran and mustard. Oxidative stability is one of the main quality constraints of vegetable oils and addressing lipid deterioration due to oxidation and resultant loss of quality and rancidity is a major challenge to the oil industry [1]. Oil bodies or lipid droplets (LDs) in the cytosol are the subcellular storage compartments of seeds comprising of the hydrophobic core of neutral lipids comprising of TAGs and enclosed by phospholipid monolayer [2, 3]. These nutrient reservoirs act as pillars of seed endurance during drought and desiccation while providing nutrients to plants during germination and post-germination growth when the seedling is incapable of photosynthesis [4]. The LD function is determined by its proteome and it is estimated that around 200 proteins are associated regardless of species [5]. However, these proteomes are highly dynamic in nature and vary widely from seed to seedling as reported in *Arabidopsis thaliana* [6]. LD formation as a seed response to dehydration and drought is very ancient and is reported as pre-dated to seed development itself [2]. The integrity and stability of LDs in plants is dependent on oleosins which are the major seed proteins and their degradation is crucial for TAG synthesis. Interestingly, the genes acting on LDs during germination resulting in TAG catabolism and lipid oxidation resulting in enhanced oil rancidity and reduced oxidative stability are similar. Both processes involve cumulative action genes including lipoxygenases, TAG lipases, phospholipases and oleosins [3, 7, 8]. LD is synthesized in the Endoplasmic Reticulum (ER) and further transported to cytosol solely defined by the innate properties of oleosin. TAG lipase is bound to peroxisomes with the help of protein complex, core retromer and later peroxisomes redirect TAG lipase to LD through tubular extensions. The other LD-associated proteins like lipoxygenases and phospholipases do not have a functional relationship with LD. However, due to their presence in the cytosol, they use LD surfaces for further actions in response to different environmental cues at diverse subcellular locations [9].

Lipoxygenases are widely distributed across the plant kingdom and bring about hydroperoxidation of polyunsaturated fatty acids (PUFAs). LOX activity on lipid degradation of stored seed reserves leads to the production of jasmonic acid and green leaf volatiles (GLVs) important in many physiological processes from germination to senescence and stress resistance [10]. Moreover, LOX activity is reported to cause undesirable ‘beany flavors’ in soybean (*G. max*) with *Glyma.13g347500 (GmLox2)*, being the main culprit [11]. At low-temperature conditions increased LOX activity is associated with phospholipase D (PLD) action in the degradation of phospholipids resulting in the release of PUFAs abundantly [12]. The intermediate products of the LOX pathway will lead to off-flavors affecting oil quality as in rice bran oil [8, 13] and oleosin, TAG lipases, PLD and LOX are the major players other than FAD2 in determining oil rancidity. PLD also function in the deterioration of phospholipid membrane on the surface of oil bodies. Like LOX, the functions of PLD include maintenance of seed quality and viability in Arabidopsis [14]; germination, stress tolerance, nutrient uptake and root system architecture development [15]. Oleosins are important for OB stabilization during the desiccation of seed and pollen, seed viability maintenance and lipid solvency during germination [16, 17]. Moreover, oleosins are directly involved in seed oil accumulation as reported in *B. napus* and *A. thaliana* [18, 19]. In addition, caleosins (CLO/PXG) the calcium binding oil surface body protein [20], steroleosins (HSD) known as sterol dehydrogenases [21] and SEIPINs encoding integral membrane proteins are crucial for LD biogenesis and organization in close association with oleosins [22]. TAG lipases like SDP1 are actively involved in germination through lipid degradation while silencing of the gene leads to increased oil content [23].

Moreover, an enhanced LD proliferation is reported along with environmental stress response and environmental adaptation in crop plants [24] resulting in overhauling the lipid metabolism. Conversely, the LD breakdown during seed germination provides energy and fuel to germinating seed till the establishment of photosynthesis [25]. The important yet contrasting functions exerted by LD in plants indicate the existence of well-defined machinery in plants critical to survival. As LDs are involved in the manifestation of fatty acid composition and enhancing the total seed oil content, the manipulation of LD packaging can lead to the production of high-value fatty acids with industrial importance [7]. Besides, the LD-associated genes like LOX, PLD, OLE and TAG lipases create a burden on the oil industry through rancidity development. The present study aims at gathering the genomics, proteomics and transcriptomics data including expression at different stages and tissues as well as the sub-cellular localization of the aforementioned classes of genes to understand evolutionary divergence in major oil seed crops. The evolutionary tradeoffs and functional divergence of LD-associated genes and orthologs in major oilseed crops domesticated and bred for higher oil yield will be beneficial to address burning issues in the oil industry.

## Methods

### Species and genes used in study

The oil-bearing plants selected for the study included both monocots and dicots and covered all the major oilseed crops. This included seven diploids, three polyploids and a partially diploidized tetraploid; soybean. The details of genes mined from each species are outlined in Table-1 whereas the taxonomic position of species used is detailed in Supplementary Information (SI-1).

### Data sources and retrieval

From the *A. thaliana* genome from The Arabidopsis Information Resource (TAIR) available at https://www.arabidopsis.org/ the CDS, protein and gff sequences were downloaded corresponding to reported lipoxygenase, phospholipase D, oleosin TAG-lipases, steroleosin, caleosin and seipin homologs. Using these sequences as querry, the homologous sequences from *Oryza sativa, Olea europaea, Gossypium hirsutum, H. annuus* and *R. communis* were retrieved from Phytozome v13 available at https://phytozome-next.jgi.doe.gov/ using TBLASTX and the *Elaeis guineensis* (oil palm) genes were retrieved from NCBI-genome. Similarly, information was obtained from Soybase (https://www.soybase.org/) for *G. max*; PeanutBase (https://www.peanutbase.org/) for *A. hypogaea* and The Brassica Database (BRAD, http://brassicadb.cn/) for *B. juncea* while for *S. indicum*, the sequences were downloaded from sesame pan-genome [26] for Zhongzhi13 and from the improved Baizhima genome assembly [27]. The latest whole genome assemblies for all the studied species were also retrieved from the above-mentioned sources. The conserved domains in the CDS were identified using NCBI-CDD database search tool [28] and pseudogenes were removed. ‘ProtParam’ tool (https://web.expasy.org/protparam/) was used for estimating different protein parameters. The ‘gff’ files were processed with MS-excel and exon numbers were calculated. The R-package ggplot2 was used for data visualization.

### Phylogenetic analysis

Bayesian phylogenetic tree was constructed for each family using the protein sequences using MCMC method using BEAST v2.6.6 [29]. Multiple sequence alignment was created using CLUSTAL X v2.1 and used to generate input ‘XML’ files using BEAUti interface [30] with the specifications as follows; model ‘GTR + I + G’, ‘Yule speciation process’ and strict clock model. Two independent runs of 10,000,000 generations of MCMC chains were produced and sampled after every 5000 generations. The files were combined with TRACER v1.7.1 [31] and the plotted posterior estimates were inspected. After discarding the first 10,000 trees as burn-in, the rest of the samples were summarized in a maximum clade credibility tree using TreeAnnotator v. 2.6.6. The final tree was drawn using FigTree ver.1.4.4 (http://tree.bio.ed.ac.uk/software/figtree/) with median heights and different classes were depicted as per available literature.

In addition, phylogenetic analysis to identify the homologs in Zhongzhi13 and improved Baizhima genomes was carried out using protein sequences with the help of NJ algorithm in MEGA X [32] using the Jones-Taylor-Thornton distance matrix with 500 bootstrap replications.

### McDonald and Kreitman analysis (MK test)

From the MCMC phylogeny tree, the members of individual clades were tested for adaptive evolution through the integrative McDonald and Kreitman test (iMKT) web portal [33] available at https://imkt.uab.cat/. The CDS sequences after removing the stop codons were aligned using CLUSTAL X v2.1 and were subjected to the MK test. The test relies on parameters; ω (Ka/Ks) and α (proportion of substitutions fixed by positive selection) while the significance of the test was carried out through Fisher’s exact test (P value). In addition, the CDS sequences were also used for conducting Tajima’s Neutrality Test [34] using MEGA-X.

### Functional annotation and protein- protein interactions

GO enrichment analysis of proteins from all four families was conducted with the help of g: Profiler (https://biit.cs.ut.ee/gprofiler/gost) web server using *G. max* genes. The protein sub-cellular localization was carried out for all the studied proteins with the help of WoLF PSORT tool (https://wolfpsort.hgc.jp/). Protein-protein interaction network was visualized with the help of STRING ver. 11.5 (https://string-db.org/). The cis-element identification was done by subjecting upstream 2000 bp promoter sequences from the start codon of all the genes from *O. sativa, G. hirsutum, A. thaliana* and *R. communis* genomes to PLANT CARE (https://bioinformatics.psb.ugent.be/webtools/plantcare/html/) and comparing with the reported Arabidopsis cis-elements.

### Synteny and collinearity

The synteny and collinearity between the genomes were studied with MCScanX using TB tools [35] and the dual synteny plots were drawn. The results of MCScanX synteny studies were further utilized for the classification of individual genes as WGD or segmental duplicates.

### Tissue-specific expression analysis of genes

The Illumina RNA-seq data were downloaded from the NCBI-SRA (Sequence Read Archive) under accession number PRJNA875260 of *S. indicum* isolate11 cv. Baizhima genome [27]. This data set included SRA data of 5 tissues (seed, stem, flower, husk and leaf) under accession numbers SRR21372823, SRR21372824, SRR21372825, SRR21372826 and SRR21372827 respectively. In addition, the SRA data of apical meristem from *S. indicum* var. Goenbaek was retrieved from accession number PRJNA810203 under SRR18210334 and included in the study. The Goenbaek genome was assembled using the improved Baizhima genome as the reference and the homologous gene expression information was utilized for study. Similarly, the RNA-seq data from *G. hirsutum* tissues including seed (SRR13834354), leaf (SRR19782156), stem (SRR19782150), flower bud (SRR24677352), cotton ball (SRR5241446) and meristem (SRR8878535) were retrieved. The RNA-seq data from tapetum cells were specifically used for studying the expression of oleosin genes from datasets SRR13073864 and SRR13073867 from *A. thaliana*. Tissue-specific expression of LOX genes was studied in the germinating cotyledons of *G. max* using data from SRR447748.

The SRA data analysis was performed by using the Galaxy online workflow [36]. The downloaded data in FASTQ format were subjected to quality checks and subjected to adapter trimming using Trimmomatic V 0.38.1. Good quality reads were aligned with Hisat2 software (http://ccb.jhu.edu/software/hisat2/index.shtml) and were further assembled with Cufflinks and further used to estimate their abundances in different RNA-Seq samples as FPKM values. Heat maps of differentially expressed genes in all the tissues were generated using the FPKM values with the ggplot2 package in R-studio (v 4.1.1).

## Results

### Lipoxygenases (LOX) in oilseeds

#### Gene structure and protein characteristics

**Fig. 1:**
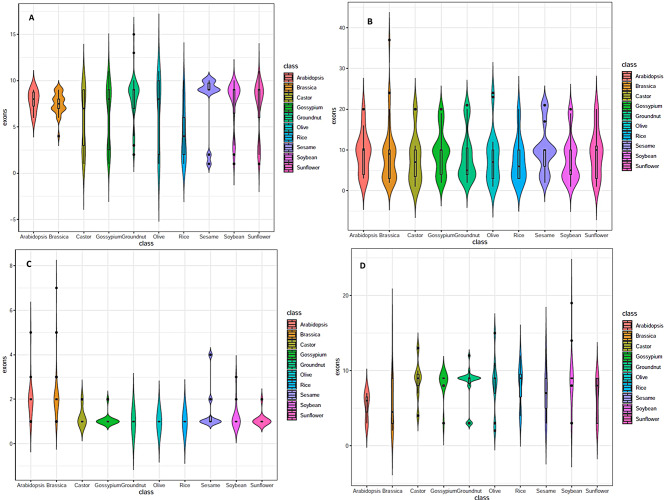
Violin plot representing the relationship between exon numbers and species with interquartile range distribution for gene families studied including lipoxygenases (**A**), phospholipase D (**B**), oleosin (**C**) and TAG-lipases (**D**)

The majority of LOX genes contained multiple exons wherein genes with 9 exons were the most common. Single exonic genes accounted for only 5.2% of the total. All the genes detected in Arabidopsis, *B. juncea* and groundnut contained multiple exons. The maximum number of exons detected for a single gene was 15 for *arahy.Y6Q89P* in groundnut. The gene *arahy.Y6Q89P* had the longest protein of 1491 AA long and with a molecular weight of 171 kDa while the shortest LOX gene detected was *Glyma.08G189100* in soybean with a protein length of 81AA long. The violin plot representing the relationship between exon numbers and species with interquartile range distribution is given in Fig. 1A. The details of individual genes are given as SI-2 A and SI2-a.

#### Phylogeny of LOX genes

The phylogeny of LOX genes was reconstructed using the multiple sequence alignment results of CDS sequences using the already reported Arabidopsis as well as groundnut classification as the benchmarks. The sequences were broadly divided into seed linoleate 9S-LOX and 13S-LOX (SI-3) based on Arabidopsis LOX genes and into Type1 and 2 based on groundnut classification reported earlier. The existence of three predominant lineages representing 9S-LOX as well as 13S-LOX (Type-1 and 2) with Type1 comprising both 9S and 13S genes was evident from the Bayesian phylogeny tree. Moreover, each lineage contained several well- defined clusters for individual species representing extensive gene duplications, especially in polyploid species like *G. hirsutum*, *B. juncea* and the *A. hypogaea*. This along with the clear separation of monocot and dicot lineages is an indication of independent evolution of LOX genes in plants. The existence of rice genes; *LOC_Os05g28770* and *LOC_Os03g08220* and their homologs from *E. guineensis* (*LOC105056718*, *LOC105041807* and *LOC105053112*) together with dicot counterparts in 13S-Type1 and Type2 lineages respectively indicate the possibility of orthologs with similar functions. The majority of *B. juncea* LOX genes belonged to 13S- Type1 sub-class while none could be detected from 13S-Type2 sub-class. The subcellular localization of 9S and 13S-LOX proteins was reported as cytoplasm and chloroplast respectively and the predicted results are given in SI-4 A.

**Table 1:** Details about oilseed species used in the study including their ploidy level, common name, family, chromosome number and the number of genes mined for each of the family associated with lipid droplets

Species	Common name	Family name	Chromosome number	LOX	PLD	OLE	TGAL	HSD	CAL	SEP	TOTAL	
*Arabidopsis thaliana*	Arabidopsis	Brassicaceae	2n = 2x = 10	6	12	18	2	8	8	3	57	
*Arachis hypogaea*	Ground nut	Fabaceae	2n = 4x = 40	48	42	3	22	8	11	6	140	
*Brassica juncea*	Mustard	Brassicaceae	2n = 4x = 36	28	39	55	16	15	20	9	182	
*Glycine max*	Soy bean	Fabaceae	2n = 2x = 40	51	29	13	17	5	6	4	125	
*Gossypium hirsutum*	Upland cotton	Malvaceae	2n = 4x = 52	51	41	26	18	9	19	5	169	
*Helianthus annuus*	Sunflower	Asteraceae	2n = 2x = 34	32	25	12	9	11	9	5	103	
*Olea europaea*	Olive	Oleaceae	2n = 2x = 46	15	23	7	10	4	6	5	70	
*Oryza sativa*	Rice	Poaceae	2n = 2x = 24	27	16	6	6	8	9	2	74	
*Ricinus communis*	Castor	Euphorbiaceae	2n = 2x = 20	21	15	5	7	4	2	2	56	
*Sesamum indicum*	Sesame	Pedaliaceae	2n = 2x = 26	10	14	8	6	4	2	5	49	
*Elaeis guineensis*	Oil palm	Arecaceae	2n = 2x = 32	12	16	9	8	5	6	2	58	
			Total	301	272	162	121	81	98	48	1083	

In addition to the reported localization sites around 30% of *O. sativa* LOX proteins were found to localize at extracellular, plasmid and peroxisomes

### Evidence of positive selection among LOX orthologs

McDonald and Kreitman analysis (MK test) was employed to detect adaptive evolution in accordance with species divergence since it is less sensitive to demographic factors like migration. The MK test can be used to detect positive selection among protein sequences even when purifying selection is operating (ω or Ka/Ks < 1). Here we used the MK test to analyze signals of positive selection among ortholog clusters inferred from the Bayesian MCMC tree. Out of the 301 LOX genes included in the study 124 sequences contained enough polymorphism to conduct the MK test. Moreover, 54 LOX genes exhibited statistically significant signatures of positive selection with a P-value < 0.05. Strong positive selection was found at 13S-LOX orthologs of sesame, olive and sunflower with both ω and α being positive. Similar case for 9S-LOX genes were noticed for rice, oil palm, castor, soybean, upland cotton and a few of the groundnut genes (Table-2). Moreover, among the fifteen 9S-LOX genes detected in rice 10 were found to exhibit strong positive selection. In sunflower, > 50% of Type-1 and Type-2 13S LOX genes belonging to were under strong positive selection. Moreover, in groundnut > 80% of the 9S-LOX genes were characterized by α being negative indicating the presence of deleterious recessive alleles under selection.

**Table 2:** Selection effects on families and orthologs as inferred through the McDonald and Kreitman analysis (MK test) of individual clades belonging to all the four LD-associated families included in the study

Cluster number	Genes	Class	Ka/Ks	α	*P*-value	Selection effect
	Lipoxygenase					
1	Gohir.A08G210200	9 S-LOX	3.047	0.853	0.002	Positive
	Gohir.D08G227100	9 S-LOX				
	Gohir.A08G210400	9 S-LOX				
	Gohir.D08G227200	9 S-LOX				
	Gohir.A08G210150	9 S-LOX				
	Gohir.D08G227051	9 S-LOX				
2	Gohir.A13G108000	9 S-LOX	2.018	0.811	0.002	Positive
	Gohir.D13G111000	9 S-LOX				
	30,128.m008781	9 S-LOX				
	Gohir.A08G210150	9 S-LOX				
	Gohir.D08G227051	9 S-LOX				
3	Oeu031399	13 S-LOX	5.875	0.938	< 0.001	Positive
	Oeu051804	13 S-LOX				
	Oeu032160	13 S-LOX				
	Zhongzhi13_32243	13 S-LOX				
	HanXRQChr15g0470751	13 S-LOX				
	HanXRQChr06g0169411	13 S-LOX				
	HanXRQChr10g0289401	13 S-LOX				
	Oeu056594	13 S-LOX				
	SIN_1005154	13 S-LOX				
4	Glyma.07G007000	9 S-LOX	1.312	0.717	< 0.001	Positive
	Glyma.07G006750	9 S-LOX				
	Glyma.07G006900	9 S-LOX				
	Glyma.07G034800	9 S-LOX				
	Glyma.07G034900	9 S-LOX				
	Glyma.08g189500	9 S-LOX				
	Glyma.08G189300	9 S-LOX				
5	LOC_Os03g52860	9 S-LOX	0.798	0.5500	< 0.001	Purifying
	LOC_Os03g49350	9 S-LOX				
	LOC_Os03g49260	9 S-LOX				
	LOC_Os03g49380	9 S-LOX				
	LOC_Os11g36719	9 S-LOX				
6	arahy.0TX3JS	9 S-LOX	0.774	0.5480	< 0.001	Purifying
	arahy.GC5CXU	9 S-LOX				
	arahy.KS8M6W	9 S-LOX				
	arahy.ZS7YSR	9 S-LOX				
	29680.m001710	9 S-LOX				
7	Oeu055817.1	13 S-LOX	0.984	0.5910	< 0.001	Purifying/Neutral
	HanXRQChr13g0404321	13 S-LOX				
	HanXRQChr13g0404271	13 S-LOX				
	HanXRQChr13g0404281	13 S-LOX				
	HanXRQChr13g0404291	13 S-LOX				
	HanXRQChr02g0056751	13 S-LOX				
	HanXRQChr02g0056761	13 S-LOX				
	HanXRQChr13g0404381	13 S-LOX				
	HanXRQChr13g0404341	13 S-LOX				
8	Glyma.13G030300	13 S-LOX	0.981	0.6220	0.001	Purifying/Neutral
	Glyma.13G075900	13 S-LOX				
	Glyma.20G053700	13 S-LOX				
	Glyma.20G054000	13 S-LOX				
	Glyma.20G054100	13 S-LOX				
	Gohir.D05G070100	13 S-LOX				
	Gohir.A05G067300	13 S-LOX				
	Gohir.D08G087450	13 S-LOX				
	**Phospholipase D**					
1	LOC_Os08g31060	PLD-alpha	2.028	0.503	< 0.001	Positive
	LOC_Os03g27370	PLD-alpha				
	LOC_Os05g07880	PLD-alpha				
	LOC_Os09g25390	PLD-alpha				
	LOC_Os06g40190	PLD-alpha				
	LOC_Os06g40180	PLD-alpha				
	LOC_Os06g40170	PLD-alpha				
	arahy.6051AB	PLD-alpha				
2	Oeu045138.7	PLD-alpha	1.01	0.776	< 0.001	Neutral
	Oeu045129.1	PLD-alpha				
	Oeu045144.1	PLD-alpha				
	Oeu042105.1	PLD-alpha				
	Zhongzhi13_06936	PLD-alpha				
	Zhongzhi13_01853	PLD-alpha				
	Oeu033458.1	PLD-alpha				
3	HanXRQChr10g0293451	PLD-zeta	1.222	0.502	0.067	Positive
	HanXRQChr13g0413971	PLD-zeta				
	HanXRQChr03g0083791	PLD-zeta				
	HanXRQChr07g0199221	PLD-zeta				
	Oeu043326	PLD-zeta				
	Zhongzhi13_24646	PLD-zeta				
4	Oeu025074	PLD-beta	0.421	0.225	0.001	Purifying
	Oeu062006	PLD-beta				
	Oeu024425	PLD-beta				
	Oeu024427	PLD-beta				
	Zhongzhi13_02558	PLD-beta				
	30174.m008942	PLD-beta				
	30190.m011102	PLD-beta				
	Gohir.D08G196000	PLD-beta				
	Gohir.A08G177600	PLD-beta				
5	Oeu026118	PLD-delta	0.633	0.554	0.001	Purifying
	Oeu007386	PLD-delta				
	Zhongzhi13_07814	PLD-delta				
	Zhongzhi13_16584	PLD-delta				
	HanXRQChr13g0387511	PLD-delta				
	HanXRQChr14g0428501	PLD-delta				
6	28694.m000682	PLD-epsilon	0.838	0.559	0.08	Purifying
	Gohir.D03G056600	PLD-epsilon				
	Gohir.A02G115700	PLD-epsilon				
	Oeu026744	PLD-epsilon				
	Zhongzhi13_06287	PLD-epsilon				
	AT1G55180	PLD-epsilon				
7	28725.m000311	PLD-delta	0.493	0.328	0.073	Purifying
	Gohir.A12G027500	PLD-delta				
	Gohir.D12G026900	PLD-delta				
	LOC_Os07g15680	PLD-delta				
	LOC_Os03g62410	PLD-delta				
8	arahy.I1DXP3	PLD-zeta	0.346	0.64	0.064	Purifying
	Glyma.15G152100	PLD-zeta				
	arahy.HZZY15	PLD-zeta				
	arahy.M1WUGI	PLD-zeta				
	**Oleosin**					
1	HanXRQChr07g0205601	OLE-SL	1.905	0.823	< 0.001	Positive
	HanXRQChr14g0460941	OLE-SL				
	HanXRQChr05g0138621	OLE-SL				
	HanXRQChr08g0223251	OLE-SL				
	HanXRQChr17g0549281	OLE-SL				
2	HanXRQChr08g0223251	OLE-SL	1.631	0.833	< 0.001	Positive
	Zhongzhi13_14219	OLE-SL				
	Oeu006820.1	OLE-SL				
	Oeu006822.1	OLE-SL				
3	Gohir.A05G254400	OLE-SH	2.569	0.848	0.004	Positive
	Gohir.D05G256000	OLE-SH				
	Glyma.16G071800	OLE-SH				
	Glyma.19G063400	OLE-SH				
	Glyma.17G086400	OLE-SH				
	HanXRQChr16g0504661	OLE-SH				
	Zhongzhi13_05546	OLE-SH				
4	AT3G01570	OLE-SH	1.761	0.754	0.026	Positive
	29917.m001992	OLE-SH				
	30147.m013891	OLE-SH				
	Oeu063474.1	OLE-SH				
	Zhongzhi13_23921	OLE-SH				
	Zhongzhi13_32113	OLE-SH				
5	Zhongzhi13_23921	OLE-SH	0.882	0.588	0.01	Purifying
	HanXRQChr17g0548541	OLE-SH				
	HanXRQChr15g0475351	OLE-SH				
	HanXRQChr14g0450531	OLE-SH				
	**TAG-lipase**					
1	Gohir.D07G194600	TGL	0.946	0.74	0.007	Purifying/Neutral
	Gohir.A07G187800	TGL				
	29620.m000557	TGL				
	Glyma.02G267100	TGL				
	arahy.X4UQJ0	TGL				
	arahy.N2VKUZ	TGL				
2	arahy.JS3G8E	TGL	2.516	0.475	0.055	Positive
	Oeu011757	TGL				
	Oeu013645	TGL				
	Zhongzhi13_32047	TGL				
	HanXRQChr15g0486941	TGL				
3	arahy.V45S4S	TGL	2.334	0.843	0.004	Positive
	arahy.DY4FLS	TGL				
	arahy.UI4NB4	TGL				
	arahy.IGHX33	TGL				
	arahy.C6629Z	TGL				
4	LOC_Os01g55650	SDP	0.945	0.68	< 0.001	Purifying/Neutral
	LOC_Os03g59620	SDP				
	HanXRQChr13g0395891	SDP				
	HanXRQChr01g0008241	SDP				
	HanXRQChr06g0176801	SDP				
5	Gohir.A13G002700	SDP	1.192	0.815	0.005	Positive
	Gohir.D13G002600	SDP				
	28470.m000422	SDP				
	arahy.92S4AT	SDP				
	arahy.K52S6H	SDP				
	Glyma.10G105200	SDP				
	arahy.E4P4QB	SDP				
	arahy.ZN271A	SDP				
	Glyma.03G130900	SDP				
	Glyma.19G132900	SDP				
	Zhongzhi13_08222	SDP				
6	BjuOB03G07420	TGL	5.253	0.919	< 0.001	Positive
	Oeu024322.1	TGL				
	Zhongzhi13_06569	TGL				
	HanXRQChr09g0269901	TGL				
	HanXRQChr05g0152881	TGL				
7	30174.m008713	TGL	1.311	0.73	0.033	Positive
	arahy.Q237M7	TGL				
	arahy.11NJV3	TGL				
	Glyma.05G232700	TGL				
	Glyma.08G040100	TGL				
	arahy.5B91PZ	TGL				
	arahy.SXG9J3	TGL				
	Glyma.07G089400	TGL				
	Glyma.09G187400	TGL				
8	Gohir.D07G174700	TGL	0.441	-1.084	0.014	Purifying
	Gohir.A07G168400	TGL				
	Gohir.D07G174600	TGL				
	HanXRQChr12g0373721	TGL				

The inference was made based on parameters ω or Ka/Ks and the proportion of substitutions fixed by positive selection (α). Significance test was done using Fisher’s exact test and here only the clusters with significant the P-values are given

**Fig. 2:**
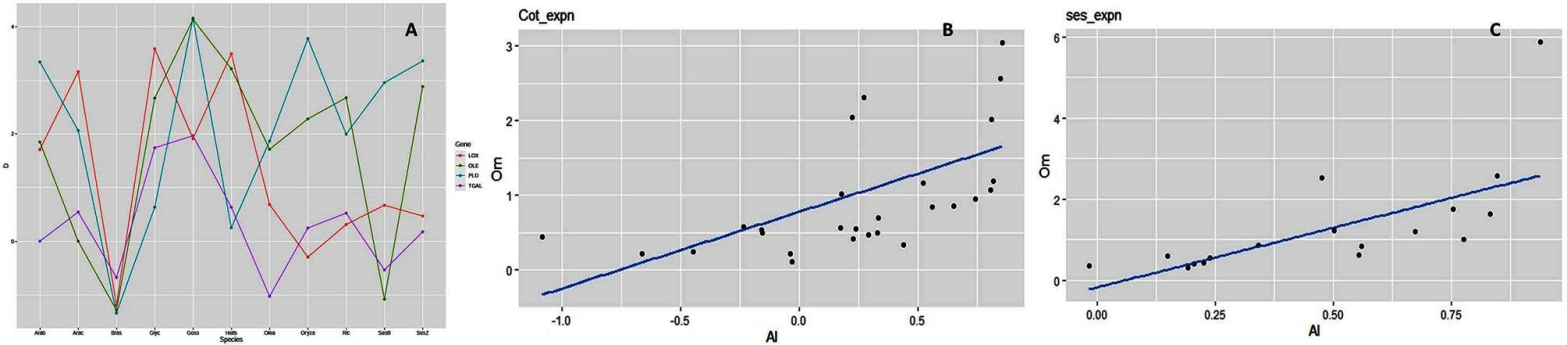
The effect of natural selection on the genes studied as indicated by Tajima’s D test (**A**) in all the species studied for lipoxygenases, phospholipase D, oleosin and TAG-lipases included in the study. The relationship between McDonald and Kreitman analysis (MK test) and the highly expressed genes of all the 4 gene families in cotton (*G. hirsutum*) (**B**) and sesame (*S. indicum*) (**C**)

Such negative α values were also evident in the ortholog clusters involving ground nut, cotton and castor as well as in lineage-specific clades of sunflower and ground nut. The MK test results indicated that in *A. thaliana* and *B.juncea* both belonging to Brassicaceae family, none of the LOX genes were under selection pressure whereas in ground nut > 64% LOX genes showed selection effects. In oil palm the 9S-LOX genes were under positive and the 13S-LOX type-II genes were under neutral selection pressures. We also used Tajima’s to detect selective sweeps in coding regions wherein all the studied loci in *B. juncea* reported negative D values indicating positive selection. However, in sunflower and cotton D values were highly positive indicating an excess of common alleles leading to balancing selection. Large variation was detected in D values between two varieties (Zhongzhi13 and Baizhima) of the same species, sesame. Details are in Fig. 2A, SI-5 A, SI2- f.

### Functional annotation analysis of LOX genes

The functions of LOX genes were analyzed by performing GO enrichment analysis based on biological process (BP), molecular function (MF), and cellular component (CC) classes with g: Profiler web interface using soybean LOX genes. Under the MF category 11 GO terms were found to be significantly enriched including GO: 0016702 (oxidoreductase activity, acting on single donors with incorporation of molecular oxygen, incorporation of two atoms of oxygen) and GO: 0046872 (metal ion binding). The GO term GO: 1,990,136 (linoleate 9 S-lipoxygenase activity) was accounted in 4 genes including *Glyma.07G007000* showing strong positive selection. GO: 0031407 (oxylipin metabolic process) and GO: 0034440 (lipid oxidation) under the category BP and GO: 0005737 (cytoplasm) under CC category were significantly enriched. Jasmonic acid biosynthesis and metabolism, lateral root morphogenesis and response to stress were particularly enriched among *A. thaliana* genes. The KEGG pathways, KEGG: 00591 (Linoleic acid metabolism) and KEGG: 00592 (alpha-Linolenic acid metabolism) were found to be significantly enriched for 28 and 15 genes respectively. Details are in SI-6 A.

### Expression profile of LOX genes in different tissues

**Fig. 3:**
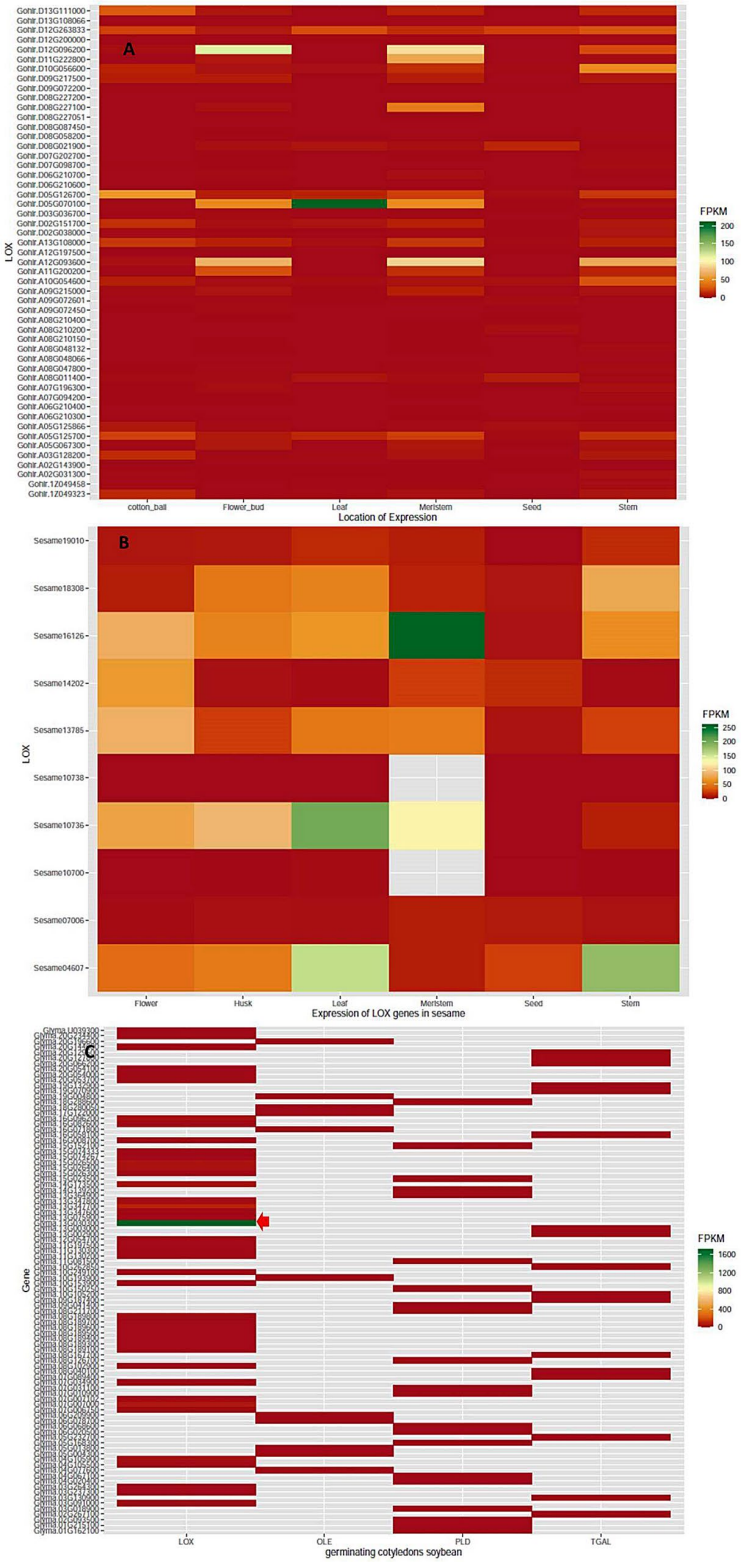
The expression of lipoxygenase genes in ball, flower bud, leaf, meristem, stem and seed in cotton (*G. hirsutum*) (**A**); seed, husk, flower, stem, leaf and apical meristem in sesame (*S. indicum*) (**B**); and the germinating cotyledons of soybean (*G. max*) (**C**). The red arrow indicates the higher expression of 13S-LOX gene, *Glyma. 13G030300* in soybean germinating cotyledons

We studied the expression profiles of LOX genes in seed, husk, flower, stem, leaf and apical meristem in sesame and in the ball, flower bud, leaf, meristem, stem and seed in cotton (*G. hirsutum*) as well as the germinating cotyledons of soybean. We used the RNS-seq data of the sesame variety ‘Baizhima’ for expression analysis and the homologs identified in the refseq variety ‘Zhongzhi-13’ is given as SI-7. A comparison of LOX gene expression in different tissues of sesame and cotton is given in Fig. 3 and SI-8 A. In sesame maximum expressions were observed in meristem, leaf and stem while in cotton the preferred sites were leaf, meristem, flower bud, stem and cotton ball. Maximum expression for a single gene was for *Sesame.16126* (13S-LOX-Type I) gene which was homologous to *SIN1005154* at meristem. Moreover, this gene exhibited strong signals of positive selection. In cotton, the maximum expression noticed was for *Gohir.D05G070100*, belonging to 13S-LOX-Type II in leaves. Most of the 13S-LOX genes were found to express in meristem in both the species whereas 9S LOX genes expressed in meristem and stem. The expression of LOX genes in seed was very low in both species. However, in germinating cotyledons of soybean 13SLOX- Type II gene, *Glyma. 13g030300* showed a 720 fold increase in expression over 13S-LOX gene in cotton, *Gohir.D05G070100*. The LOX gene *Glyma.13g347500* associated with off-flavor was not expressed during germination, rather its paralog *Glyma.13g347600* showed expression. All the genes exhibiting positive selection showed consistent expression across tissues in cotton. The genes showing higher expression in cotton and soybean were under purifying selection with highly significant P-value (0.001).

### Phospholipase D (PLD) genes in oilseed crops

A total of 272 PLD genes were used for the study, wherein the majority was contributed by polyploid species of *G. hirsutum*, *B.juncea* and *A. hypogaea*.

### Gene structure and protein characteristics

Oilseed PLD genes were characterized by the presence of multiple exons in 98% of genes across species. Single exon genes were observed in castor, soybean and olive genomes. The maximum number of exons noticed for a single gene was 24 in Olive (*Oeu031987.1*) and two *B.juncea* genes (*BjuOB05G01770* and *BjuOA01G02960*). The most common exon number was 10. The longest protein was 2712AA long in *BjuOA01G02960* with a molecular weight of 304.6 kDa, while the shortest was 100AA long in olive (*Oeu041845.1*). Details are in Fig. 1B and SI-2B.

### Phylogeny of PLD genes

The Bayesian phylogeny tree classified the PLD genes into α, β, γ, δ, ε, ζ and ψ classes based on sequence similarity to already published *A. hypogaea, A. thaliana* and *O. sativa* PLD classification. The phylogeny represented an interesting picture where the PLD genes from ground nut, Arabidopsis, *B. juncea*, cotton, castor and rice shared greater similarities between the sequences while the other cluster belonged to sesame, olive, soybean and sunflower. In monocots, ε class members were absent while the classes δ, γ and ζ shared greater homogeneity between members. Class ψ members were present only in rice, cotton, castor and sunflower. The ψ genes retained a closer similarity to class ζ members of soybean, castor and sunflower presented several unique PLD genes with considerable sequence deviation from the represented class members. Class α was highly diversified and included maximum members followed by class δ. The representation of species *B. juncea* and *S. indicum* were much less in ζ and β classes respectively. The detailed phylogeny is given in SI-9.

### Evidence of positive selection among PLD orthologs

Significant polymorphism was noticed in 160 PLD genes and was used for the MK test. Among these 69 loci were shown signatures of positive selection with ω > 1, while α values were also positive in 63 loci. In castor and sesame > 85% of PLD genes were under selection whereas selection pressures were low in *B. juncea*, oil palm and sunflower. Maximum PLD genes were under positive selection in rice. The classes δ, ε, α and ζ were under strong selection pressure whereas maximum positive selection signals were detected in the class α. In rice almost all of the genes belonging to classes α were under positive selection while all the δ genes were under purifying selection. Except for sunflower and *B. juncea* genes, all the other ε genes were under selection which was balancing in nature. Moreover, none of the Arabidopsis PLD genes belonging to class α were under selection. However, the high positive values obtained for Tajima’D statistic indicated an excess of common alleles in most of the species. Details are in Fig. 2; Table-2 and SI-5B.

### Functional annotation analysis of PLD genes

Under the MF category 13 GO terms were found to be significantly enriched which included GO: 0004630 representing phospholipase D activity. Similarly, lipase activity, hydrolase activity and cation binding were also crucial and involved a large number of PLD genes. Under the biological processes, 22 GO terms were enriched including GO: 0009395 and GO: 0048017, representing phospholipid catabolic process and inositol lipid-mediated signaling respectively. Moreover, the ζ group members were uniquely associated with phosphatidic acid biosynthesis and metabolism in addition to inositol lipid-mediated signaling. The cellular components included the plasma membrane and cell periphery. The KEGG processes included glycerophospholipid metabolism, ether lipid metabolism, endocytosis and biosynthesis of secondary metabolites (SI-6B). The major sites of protein localization were the nucleus, cytosol and cytoskeleton. The class ψ members of PLD genes in plants were found to localize at extracellular space (SI-4B).

### Expression profile of PLD genes in different tissues

**Fig. 4:**
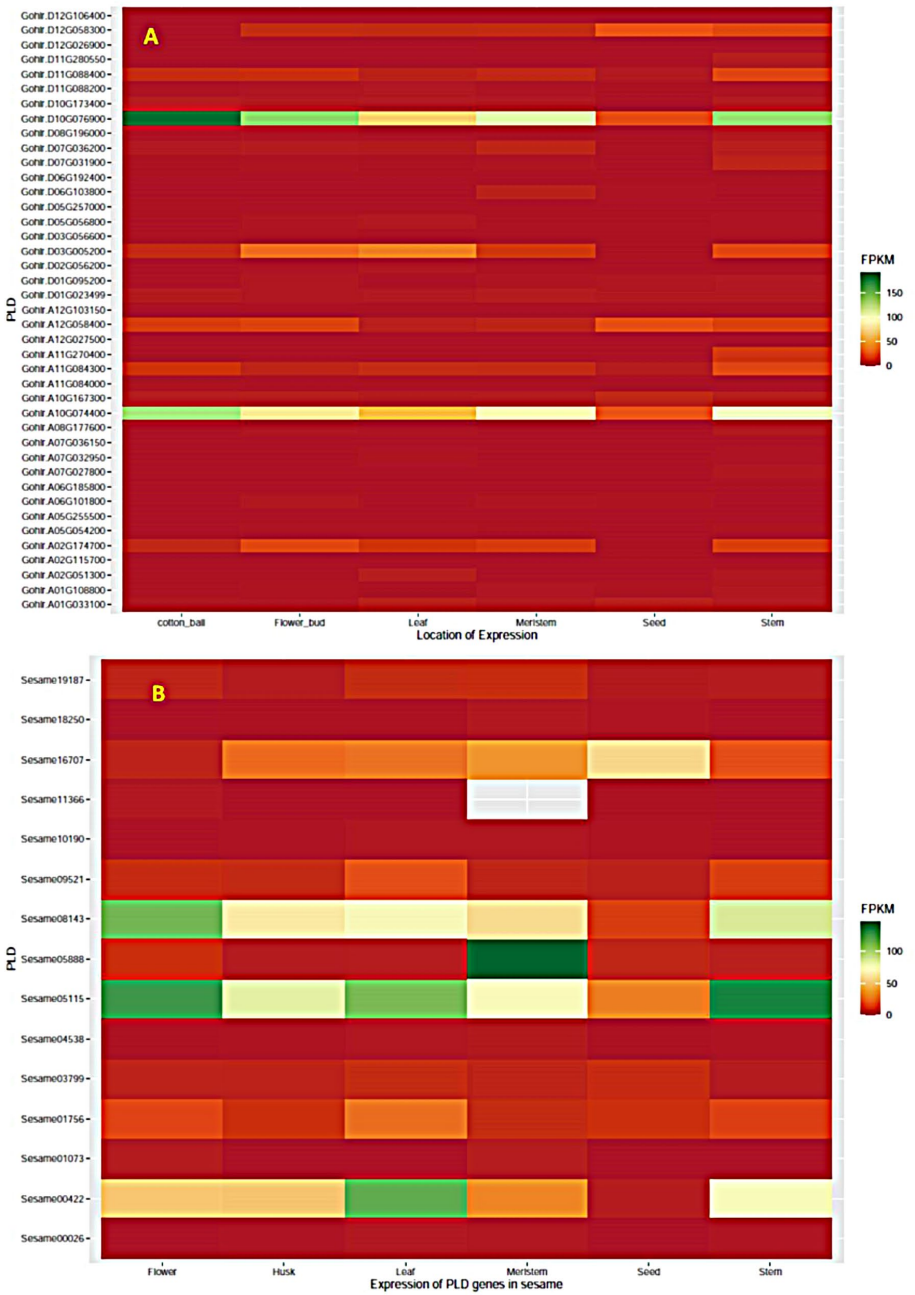
The expression of phospholipase-D genes in ball, flower bud, leaf, meristem, stem and seed in cotton (*G. hirsutum*) (**A**) and seed, husk, flower, stem, leaf and apical meristem in sesame (*S. indicum*) (**B**)

The site of expression for different classes of PLD genes differed widely across the different classes. In sesame, the PLD-α genes were expressed on all the tissues except the seed while the δ genes were expressed on the seed and meristem. A higher expression of β and γ genes was observed in the leaf (Fig. 4 and SI-8B). A similar pattern of expression was observed in cotton. In addition, the α genes showing higher expression in sesame including *Sesame.05115*, *Gohir.D10G076900* and *Gohir.A10G074400* were under strong positive selection and exhibited higher expressions across all tissues. On the contrary, another α gene, *Sesame.08143*, with similar expression patterns across tissues was under balancing selection. The homologs of sesame genes in the reference genome are *Zhongzhi13_06936* and *Zhongzhi13_11817*. The δ genes showing higher expression in sesame *Zhongzhi13_23098* (*Sesame.16707*) and *Zhongzhi13_07814* (*Sesame.05888*) were under purifying or neutral selection. However, the δ genes of cotton with high expression, *Gohir.D03G005200* and *Gohir.A02G174700* were under positive selection. Similarly, the γ genes in sesame including *Sesame.00422* (*Zhongzhi13_01075*) were under negative selection (Fig. 4).

### Oleosin (OLE) genes in oilseed crops

A total of 161 oleosin genes were used in the study where an increased copy number was encountered in Brassicaceae members, *A. thaliana* and *B. juncea*. These two species contributed 50% of the detected oleosin genes.

### Gene structure and protein characteristics

The majority of the oleosin genes were single exonic with protein lengths measuring 150-200AA. Multi-exonic genes were more common in Brassicaceae family members, to the extent of 80% whereas in all the other genomes together the extent was less than 10%. The largest and heaviest oleosin protein detected was *BjuOA02G02720* with a length of 2039 AA and a molecular weight of 203.7 kDa. The shortest protein observed was in sesame of length 90 AA in *Zhongzhi13_14219*. The maximum number of exons observed in a single gene was 7 for *BjuOB05G24980*. Strikingly, most of the oleosin proteins recorded very high isoelectric point values of > 9. The highest value observed was 11.93 for *AT5G07510* from *A. thaliana*. Details are in Fig. 1C and SI-2^2^ C.

### Phylogeny of oleosin genes

The oilseed oleosin genes were classified into Seed High-molecular weight (SH), Seed Low-molecular weight (SL), Universal (U) and Tapetum (T) lineages. The T lineage was present only in Brassicaceae members and accounted for 50% of genes detected in the family. All of the T lineage members were multi-exonic in both the species. Like the PLD genes; sesame, soybean, olive and sunflower shared greater homology with each other than the others. The monocot oleosin genes shared greater homology with each other than with the eudicots. The details are in SI-10.

### Evidence of positive selection among oleosin orthologs

102 oleosin genes exhibited significant polymorphism to qualify for the MK test. Among these 50.1% of genes had ω > 1 and a positive α, indicating the presence of strong positive selection in operation. 72% of oleosin genes in sunflower, sesame and olive were under positive selection with SL lineage having the highest share of 80%, followed by SH (75%) and U (66.7%) lineages. In *B. juncea*, 42.1% of the genes were under positive selection, including all of the SL lineage genes and 75% of the U lineage genes. However, only 31% of T lineage genes and 22% of SH lineage genes were under positive selection. The proportion of substitutions fixed by positive selection was 13.8 to 65% in *B. juncea* compared to 67.3 to 84.8% in the other group. In soybean only 38.5% of oleosin genes were under positive selection whereas the value was quite low in cotton (7%) and Arabidopsis (5.5%). All of the *A. thaliana* T lineage genes were under purifying selection. Moreover, four of the *B. juncea* oleosin genes were under neutral selection, with ω = 1. Details are in Table-2 and SI-5 C.

### Functional annotation analysis of oleosin genes

The major biological processes associated with soybean oleosin genes were lipid storage (GO: 0019915), lipid localization (GO: 0010876) and seed oil body biogenesis (GO: 0010344). In addition, the GO terms corresponding to response to stress, reproductive process and post- embryonic development were significantly enriched. The major cellular components (CC) associated were membrane, lipid droplet and intracellular non-membrane-bounded organelle. In, Arabidopsis the T-lineage oleosin genes were involved in functions mainly anther, tapetum and anther wall morphogenesis. In addition, pollen coat and extracellular matrix were found associated with cellular components. Details are in SI-6 C. Plasmid, chloroplast and vacuole were the preferred sites of protein sub-cellular localization. T-lineage genes were localized particularly in plasmid and vacuole followed endoplasmic reticulum and golgi bodies. The U-lineage genes of *G. hirsutum, A. thaliana, A.hypogaea* and *B. juncea* oleosins localized at the chloroplast while mitochondria were the preferred location for other species. Oleosins of *O. sativa* localized on plasmid, cytosol and ER while none of the *G.max*, *H. annuus*, *S. indicum* and *O. europaea* genes localized on plasmid or vacuole (SI-4 C).

### Expression profile of oleosin genes in different tissues

**Fig. 5:**
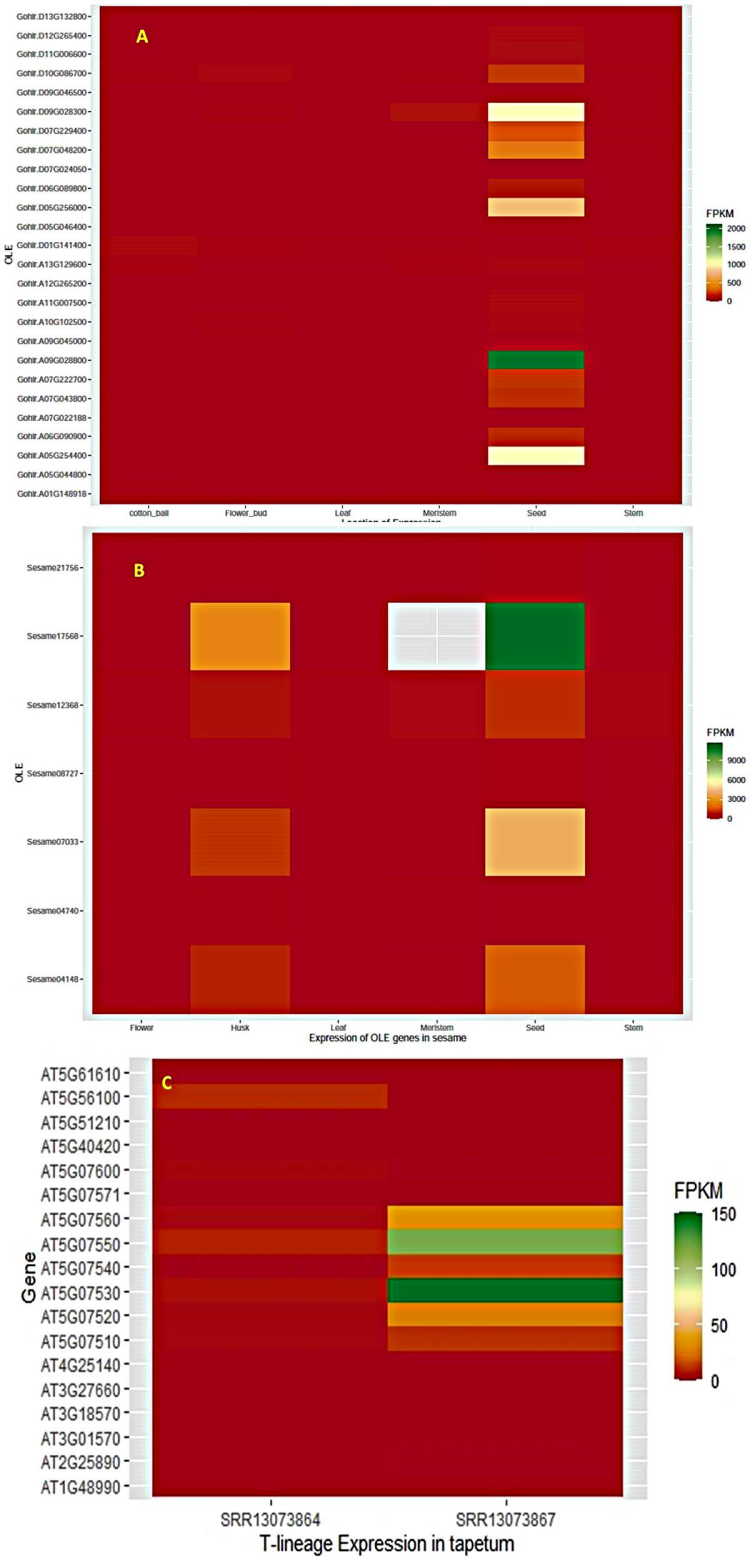
The expression of oleosin genes in ball, flower bud, leaf, meristem, stem and seed in cotton (*G. hirsutum*) (**A**) and seed, husk, flower, stem, leaf and apical meristem in sesame (*S. indicum*) (**B**) and the tapetum cells of *A. thaliana* (**C**)

Maximum expression of oleosin genes was observed in seed and husk. The SH-lineage genes *Sesame.17568* (*Zhongzhi13_23921* and *Zhongzhi13_32113*) as well as *Sesame.07033* and *Sesame. 04148* (*Zhongzhi13_05546*) were found to have the highest expression in both the tissues. Similarly, the SL-lineage gene *Sesame.12638* (*Zhongzhi13_09571*) had the highest expression in seed and husk whereas U-lineage genes consecutively expressed all the tissues. In cotton also maximum expression was observed in seed for SH and SL lineage genes. Oleosin gene expression was much higher in sesame seed compared to cotton and the fold change expression for top most ranking genes were 5.5 and 2.3 for SH and SL lineages respectively (Fig. 5). Moreover, all of the highly expressed genes were also positively selected in sesame, while in cotton one set of SH-lineage gene (*Gohir.A05G254400* and *Gohir.D05G256000*) was under positive selection. However, all the expressed T-lineage genes of Arabidopsis were under purifying selection (Fig. 2, SI-8 C).

### Triacyl glycerol (TAG) lipase genes

121 TAG-lipase genes were studied belonging to Sugar Dependent-1 (SDP1) and Triacyl Glycerol Lipase 1(TGL1) types. The MCMC phylogeny tree depicting classification is given as SI-11. SDP-1 type contained 3 exons most commonly whereas most of the TGL1 type had 9 exons. The maximum exonic number detected was 19 in *Glyma.10G262850* from soybean which was a fusion product of the TGL domain with the NHAD transporter family. Such gene fusions with exonic numbers of 10–15 were detected in other TGL genes namely *Glyma.20G129500* (NHAD transporter), *Oeu013645.3* (two TGL domains) and alpha-beta hydrolases in *Oeu018583.1, BjuOA07G29570, arahy.11NJV3* and *arahy.Q237M7*. In spite of larger exonic numbers, the TGL1 type genes were characterized by shorter proteins. The longest and heaviest protein detected was *LOC_Os01g55650* belonging to the SDP-1 category with 1045 AA and a molecular weight of 114.9k Da. Details are in Fig. 1D and SI-2D.

### Evidence of positive selection among TAG lipase orthologs

The MK test was conducted with 84 TAG lipase genes among which 49% of genes exhibited strong signals of positive selection while 22.6% were under neutral selection. 50% of *B. juncea* genes and all of the SDP-1 genes of rice were under neutral selection. In sunflower, the genes under the categories of positive and neutral selection were 3 each (33.3%). The highest amount of positive selection (64.7%) was noticed among soybean TAG lipase genes while the lowest was noticed in castor (28.6%). Details are in SI-5D.

### Functional annotation analysis of TAG lipase genes

The major GO terms associated with TAG lipase genes under the MF category are GO: 0016298 (lipase activity) and GO: 0016787 (hydrolase activity) and lipid metabolic process (GO: 0006629) under the BP category. The cellular compartment identified was lipid droplet. The KEGG processes associated are steroid biosynthesis, linoleic acid metabolism, alpha-linolenic acid metabolism and glycerolipid metabolism. The major protein subcellular localization sites were chloroplast, plasmid, vacuole and nucleus. The majority of the positively selected genes from all the genomes and the genes under neutral selection *B. juncea* were localized in plasmids. Details are in SI-4D and SI-6D.

### Expression profile of TAG lipase genes in different tissues

The location of expression of TAG lipase genes was meristem, husk, leaf and flower in sesame and seed, flower bud and leaf in *cotton*. However, the level of TAG lipase gene expression was 4–5 fold lesser than LOX and PLD gene expression in all tissues studied. A similar level of expression was noticed in *A. thaliana* tapetum cells while a very low level of expression was observed in germinating cotyledons of *G. max*. Moreover, most of the expressed genes were under purifying selection in both species except for *Zhongzhi13_08222*. Details are in SI-8D.

### Steroleosin genes (HSD) in oilseed crops

82 steroleosin genes were retrieved from the 11 species under study for further analyses (SI2-b). The most common exon number for steroleosin genes was 6. *B. juncea* and sunflower genomes contained more duplicated genes while sesame genes were more diverged compared to others. The MCMC phylogeny revealed the existence of 7 clusters wherein 5 major clusters included the homologs of Arabidopsis steroleosin genes classified as HSD1 to HSD6. HSD2 and HSD3 were highly similar and occupied a single cluster whereas the 6th cluster was formed by sesame and castor genes. The monocot steroleosin genes were highly diverged compared to dicot genes and occupied a single separate cluster. The HSD-5 genes from Arabidopsis, soybean, cotton, *B. juncea*, oil palm, sunflower, groundnut and olive were under stringent positive selection. Similarly, a positive selection response was observed in HSD-6 genes whereas the selection effects were of purifying in nature in HSD-1 and HSD-4 genes. All the details are given in SI2-h. Expression of steroleosin genes was mainly concentrated in the seed whereas for HSD-4 types meristem was also a preferred site.

### Caleosin genes (CLO) in oilseed crops

98 caleosin genes were used for the analyses and were characterized by shorter proteins of 82 to 266 amino acids in length. More than 80% of members had 6 exons in the gene structure. The MCMC phylogeny revealed clear divergence between monocot and dicot *CLO* genes as well as caleosins from castor, olive and oil palm from other oil seed crops. *CLO3, CLO1* and *CLO8* occupied single clusters whereas significant duplications were observed among *CLO4, CLO6* and *CLO5, CLO7* classes. Significant positive selection was observed in CLO1 and CLO2 classes in Arabidopsis, *B. juncea*, castor, cotton and sunflower. The *CLO3* genes belonging to the Brassicaceae family were under significant purifying selection. The caleosin genes were found to be expressed in several parts of the cotton plant including stem, leaf, meristem and flower bud whereas the highest expression was observed in fruit and seed. The Arabidopsis caleosin gene which had high expression in the pollen tissues (*AT2G33380*) was under significant purifying selection. Caleosin genes were also found to have expression in the germinating cotyledons of soybean. The details are given in SI2-h.

### SEIPIN genes in oilseed crops

48 SEIPIN homologs were identified from different oilseed crops based on similarity to Arabidopsis genes. The majority of them contained single or two exons in the gene structure and with an average length of 308 amino acids long proteins. The MCMC phylogeny tree revealed SEIPIN 2 and 3 to be closely related and form a single cluster while SEIPIN1 genes formed another cluster. The SEIPINs of castor, olive and oil palm were found to be more diverged from Arabidopsis as well as the other oil seed crops SEIPIN genes and formed a separate cluster. The SEIPIN-2 from Arabidopsis (*AT1G29760*) and its homologs from *B.juncea* and sunflower were under significant purifying selection. The major location of expression was seed and pollen whereas expression was also observed in fruit and leaf. The SEIPIN-2 gene from Arabidopsis which was under negative selection pressure has an elevated expression in the pollen tissues.

Steroleosins, caleosins and SEIPINs are associated with biological processes such as lipid metabolism (GO: 0006629) and biosynthesis as well as lipid droplet organization (GO:0034389). HSDs are mainly involved in steroid metabolism while CLOs are involved in oxylipin metabolism. The SEIPINs have a wide variety of biological roles from regulation of fertilization to seed maturity and dormancy in addition to lipid localization (GO: 0010876) and lipid droplet formation (GO: 0140042). The cellular components associated are lipid droplets, endoplasmic reticulum and membrane. The caleosins are associated with KEGG pathway (KEGG:00073) representing cutin, suberine and wax biosynthesis while having a molecular function of calcium ion binding (GO:0005509). The major sites of protein localization are the nucleus and mitochondria while caleosin proteins are localized in the chloroplast also.

### Synteny among the genomes and duplication status

**Fig. 6:**
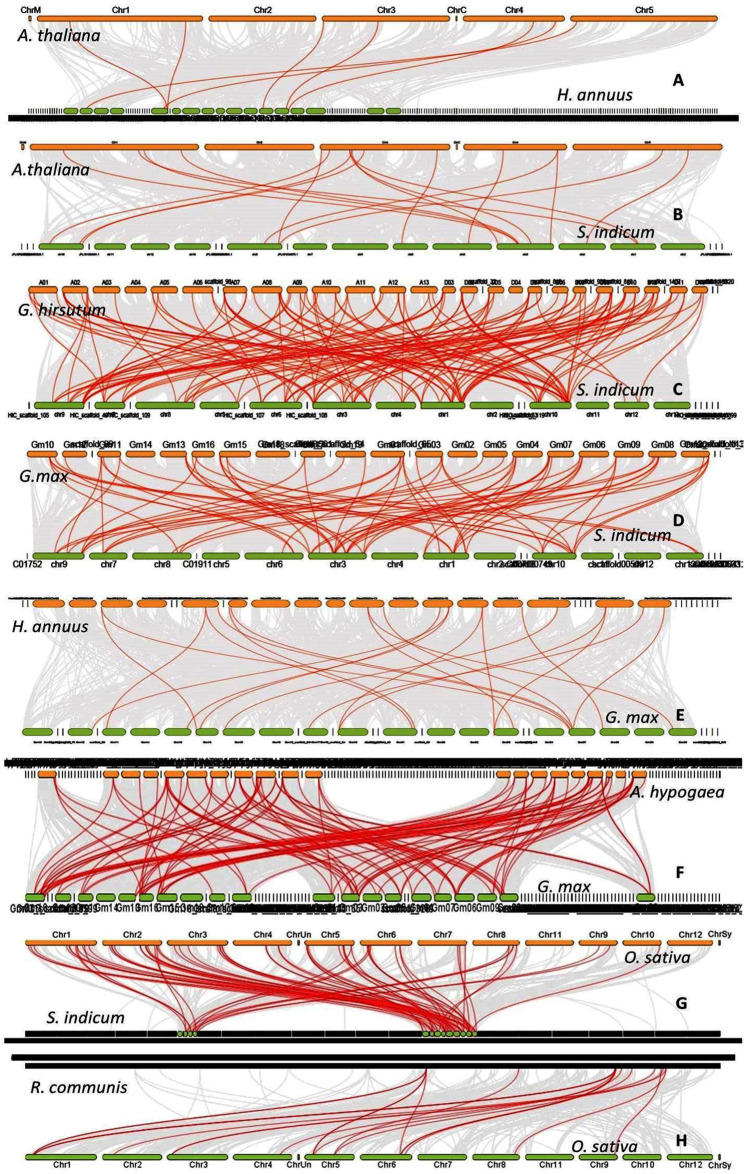
Synteny among the genomes of *A. thaliana Vs H. annuus* (**A**), *A.thaliana Vs S. indicum* (**B**), *G. hirsutum Vs S. indicum* (**C**), *G.max Vs S. indicum* (**D**), *G.max Vs H. annuus* (**E**), *A. hypogaea Vs G. max* (**F**), *O. sativa Vs S. indicum* (**G**) *and R. communis Vs O. sativa* (**H**)

**Fig. 7:**
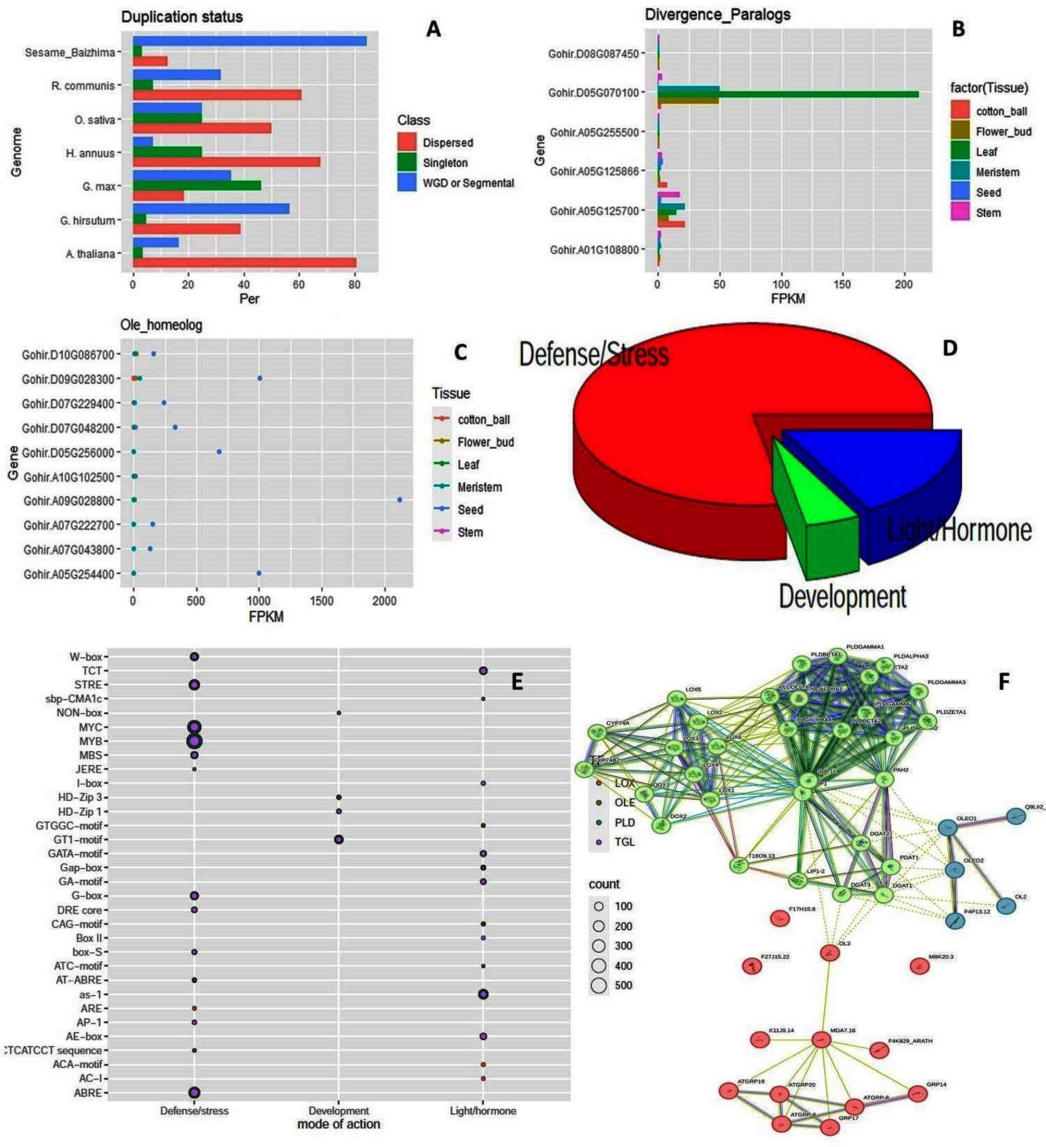
Duplication, Cis-element distribution and protein-protein interactions in the species studied. Duplication status among the genomes (**A**), divergence of paralogs as expression difference among different tissues in *G. hirsutum* (**B**), divergence of homeologs and altered expression among different tissues in *G. hirsutum* (**C**) functional divergence of cis-elements as pie-chart (**D**) Cis-element distribution in *G. hirsutum, A. thaliana, O. sativa* and *R. communis.* (**E**) and string diagram representing protein-protein interactions (**F**)

Synteny was studied among the genomes of *A. thaliana* Vs *H. annuus*, *A.thaliana* Vs *S. indicum*, *G. hirsutum* Vs *S. indicum*, *G. hirsutum* Vs *A. thaliana, G. max* Vs *S. indicum*, *G. max* Vs *H. annuus*, *A. hypogaea Vs G. max*, *O. sativa* Vs *S. indicum* and *R. communis* Vs *O. sativa* (Fig. 6). Other than the two cultivars of *S. indicum* included in the study, collinearity was found to be higher among the genomes of *A. hypogaea* and *G. max*, *G. hirsutum* and *S. indicum*, *G. max* and *S. indicum* as well as *G. hirsutum* and *A. thaliana*. The collinearity between *S. indicum* and *O. europaea* was moderate in spite of the close taxonomic position. Similar was the case of *H. annuus*. A lack of synteny was noticed with respect to chromosomes A04 and D04 in *G. hirsutum*, chromosomes 2, 11 and 13 in *S. indicum*, 14 and 18 in *G. max* and 11 and 12 in *O. sativa* with other genomes. Whole genome and segmental duplication genes were more common in *S. indicum* and *G. hirsutum* while dispersed duplicates were more common in *H. annuus*, *A. thaliana* and *R. communis*. Singleton genes were more predominant in *G. max*. (Fig. 7A). The expression divergence of paralogs in *G. hirsutum* and the differential expression of homeologs are given in Fig. 7B and C.

### Cis-element distribution among the genomes

The common cis-elements found among LOX, PLD and OLE genes were MYB, MYC, ABRE involved in defense or stress signaling, GT-1 in development and as-1 and TCT in light or hormone response. The distribution among different classes of genes and their share in different biological processes is given in Fig. 7D and E. In addition, the CLO genes contained G-box elements and the HSD genes contained as-1 and AE-1 elements with functions in light and stress response.

### Protein-protein interaction

Interplay of LOX, PLD and OLE genes was observed during seed germination by including different partners involved in Triacylglycerol lipase (TAG) metabolism and lipid hydrolysis. Notable functions include the release of fatty acids from oil bodies during germination, production of fatty acid substrate for the linolenic acid oxidation pathway and stabilization of lipid bodies during the desiccation of seed. The protein-protein interactions indicated close association of HSD4 and HSD7 and CLO4 and CLO7 with Arabidopsis oleosin genes. Similarly SEIPIN-2 and 3 had significant interactions with OLE-1 gene of Arabidopsis. Details are in Fig. 7F and SI2-Fig-4.

## Discussion

The LD-associated genes are interesting candidates to study the evolutionary divergence in plant kingdom particularly due to the crucial roles played by them in lipid metabolism associated with plant growth and development. The lipoxygenases are non-heme iron-containing enzymes that catalyze the oxygenation of fatty acids in plants. LOX genes have been categorized as 13S-LOX and 9S-LOX and are vital to plant defense through synthesis of Jasmonic acid [37, 38], production of flavor profile in plants [39–41] fruit ripening [42], biotic stress resistance [43] and germination of seed [45]. LOX activity during storage leads to enhanced peroxidation and reduced nutritional quality and vigor, especially in lipid-rich seeds like soybean, chickpea etc [44, 45]. Similarly, phospholipase D genes are also actively involved in seed germination, stress response, root system architecture and nutrient uptake, pollen tube growth, stomatal movement, rhizobium-root interactions, lipid signaling and oil rancidity [8, 15, 38, 46, and 47]. Likewise, oleosins are the LD-specific plant proteins that are involved in myriads of physiological processes like seed germination, lipid metabolism, stress response, hormone signaling, seed oil content enhancement, storage lipid stability and rancidity [13, 17, 18]. Caleosins, steroleosins are aslo included in the phospholipid monolayer of LDs in addition to oleosins [20, 21] while SEIPINS are also involved in lipid droplet organization and lipid metabolism [22] in addition to seed germination and maturity. Furthermore, TAG lipases are actively involved in seed germination, lipid metabolism, membrane lipid remodeling, stress response, LD biogenesis, and oil rancidity [48–50, and 51].

It is fascinating to note that all four classes of genes are involved in facilitating seed germination and concurrently enhancing lipid peroxidation and thereby producing rancidity and off-flavors in oil seed crops. The protein-protein interaction network clearly envisages the collective roles of LOX, PLD, OLE and TAG lipases in lipid peroxidation and associated ROS signaling. The ROS scavenging function utilizing additional genes in the peroxidase and peroxygenase pathway indicates the diverse roles of lipid droplets rather than the storehouse of energy and nutrients in seeds [5]. In *A. thaliana* it was reported that the over-expression of oleosins may increase the seed oil content [19]. The germination potential of a seed is directly linked to the oil content and a reduction of which happens during late maturity due to the action of TAG-lipases like SDP-1. The suppression of SDP-1 orthologs in *G. max* resulted in enhanced oil content as well as seed size [52]. However, the first step in germination is the removal of the oleosins through ubiquitination [53] which explains the complete lack of oleosins in the germinating cotyledons of *G. max*. Moreover, LDs serve as a sink for ROS during cellular responses during stress through membrane remodeling [5] whereas they trap fungal toxins during infection thereby reducing their ability to produce ROS [54]. Polyunsaturated fatty acids like α-linolenic acid (18:3) are more susceptible to ROS-induced peroxidation compared to oleic (18:1) and linoleic (18:2) acids explaining the role of LDs in membrane remodeling [55].The difference in the fatty acid composition of *G. hirsutum* and *S. indicum* with the latter reported to have more than double the amount of mono-unsaturated fatty acid will explain the oxidative stability [56]. A difference of 30–50 folds in the up-regulation of oleosins was noticed in the biodiesel plants jatropha and vernicia similar to the differential fold expression observed among *S. indicum* and *G. hirsutum* [57].

MK test indicated significant positive selection in the evolution and diversification of orthologs belonging to all four classes. The impact of selection in coding sequences indicated their rapidly evolving and lineage-specific behavior. Positive values of α were noticed in 60–86% of clades with the lowest value detected in LOX and highest in TAG-lipases. The α value being positive is an indication that non-synonymous variants in the genes are on the path to becoming fixed in the future [58]. Gene families showing wide variations in size and signs of positive selection are reportedly the major candidates for adaptive evolution which has been substantiated in the present study [59]. Expansion/ duplication followed by positive selection is crucial for plant adaptive evolution and multi-domain gene fusions are reported to provide adaptive advantage in stress signaling [60]. Such gene fusions and duplication of genes were evident, particularly in TAG lipases and oleosins. High levels of positive selection were associated with independent domestication in plants [61] and animals [62, 63]. Paralogs under positive selection were highly susceptible to mutations wherein evolution is directed towards diversified function [61] while fitness effects vary with different species [58]. In tetraploid cotton [64], reported an expression level dominance (ELD) bias towards the A-genome. However, in the present study such a bias was evident only in the highly expressed oleosin paralogs *Gohir.A09G028800* and *Gohir.A05G254400* whereas the bias didn’t exist in LOX, PLD and TAG lipase families. The homeologs in *G. hirsutum* showed a tissue-specific expression also wherein one was highly expressed in a particular tissue compared to the other. This was evident in *Gohir.A09G028800* and *Gohir.D09G028300* wherein the expression was higher for the A homeolog in seed whereas in meristem the D homeolog was more active. In a previous study [65], no evidence for positive selection was found among fatty acid metabolism and accumulation genes in olive although the trait is critical for domestication. In the present study, the TAG-lipases *Oeu024322*, *Oeu011757* and *Oeu013645* were found to have significant positive selection effects with a P-value < 0.05. Expression divergence was found to be significant among paralogs wherein one gene gave predominantly higher expression whereas the other gene gave a lower or nil expression in different tissues [66].

One of the classical theories of evolution is Medawar’s hypothesis about ageing [67] states that the force of selection declines with ageing. This indicates that natural selection effects tend to be higher on genes expressed early in life whereas those expressed later in life tend to accumulate deleterious mutations more [68]. In the present study, the scope of the above hypothesis was tested with the clade involving the LOX gene, *Glyma. 13G030300* in *G.max* showing higher expression during germination and its orthologs in *G. hirsutum*. The MK test indicated that the effect of purifying selection was high and it was supported by Fisher’s exact test. Such strong purifying selection effects were also visible in the highly expressed oleosin genes of *G. hirsutum*. A comparison of the coding sequences of orthologous genes detected diverse signatures of natural selection in the adaptive evolution of LDs and associated proteins. The results imply the strength and pattern of strong positive and purifying selection impact the evolution of LDs and associated proteins while emphasizing the load of deleterious mutations in lipid metabolism-associated genes arose probably due to the intensive breeding efforts to enhance the oil content. The functional divergence of LDs and associated proteins in the route to higher oil yield overhauled the lipid metabolism and storage affecting all aspects of seed life- from dormancy, germination, maturity and storage.

## Conclusions

LDs are present in all the phyla across the tree of life and are vital for cellular metabolism through coordination and interaction with different organelles (Olzmann and Carvalho, 2019). LD maintenance as well as lipophagy resulting in lipolysis during seed germination and stress response is at the opposite ends of lipid metabolism. The importance of LDs in energy homeostasis and lipid metabolism has awakened the interest of researchers, especially in the areas of cell biology and biochemistry. With the increased availability of information about genome, proteome and transcriptome in the public domain it becomes easier for researchers to trace natural selection signatures in evolution. The McDonald-Kreitman (MK) test is an excellent tool to visualize deviation from neutral evolution in the coding sequences as a result of selective pressures. The present study indicated that positive selection is the driving force behind the adaptive evolution of LD in oilseed crops. This was visible across species as we tested individual clades of orthologs for selection signatures. Moreover, most of the genes under high selection pressure are characterized by higher expression. The existence of deleterious recessive alleles with higher expression provides challenges to breeders especially while targeting heterosis. Among the paralogs, it was interesting to note that one copy was subjected to intense selection pressure and had higher expression while the other one remained neutral. The paralog divergence indicates that under the heavy influx of random mutations, the genomes are safeguarded effectively. Moreover, the effect of imprinting on these genes can be suspected. The genes identified in the present study can become suitable candidates for enhancing seed oil content and germination through artificial selection simultaneously enhancing the scope for genome editing tools. Moreover, comprehensive studies on LD biogenesis, maintenance, deterioration and assembly may unravel many cellular secrets, especially immunity, pathogenesis, and stress resilience in plants as well as the pathogenesis of many diseases in humans.

### Electronic supplementary material

Below is the link to the electronic supplementary material.

Supplementary Material 1

Supplementary Material 2

Supplementary Material 3

Supplementary Material 4

Supplementary Material 5

## Data Availability

The summarized data supporting the findings of the article is given as supplementary information. The accession numbers of each locus studied is given under the family for each species in the supplementary information. The original data links are given as URLs in the article.

## Abbreviations

LD: Lipid Droplet
LOX: Lipoxygenases
PLD: Phospholipase D
OLE: Oleosin
TAG lipase: Triacyl Glycerol (TAG) Lipase
ER: Endoplasmic Reticulum
PUFA: PolyUnsaturated Fatty Acids
SDP1: Sugar Dependent1
GLV: Green Leaf Volatiles
MCMC: Markov chain Monte Carlo method
NJ: Neighbor Joining
MK: McDonald and Kreitman test
FPKM: Fragments Per Kilobase Million
ROS: Reactive Oxygen Species
