# Supplementary material for: Exploring selection signatures in the divergence and evolution of lipid droplet (LD) associated genes in major oilseed crops
Source: BMC Genomics. 2024 Jul 1;25:653. doi: 10.1186/s12864-024-10527-4 (PMC11218257; doi:10.1186/s12864-024-10527-4)
Supplement: Supplementary file 3 — Supplementary Material 3 [file 12864_2024_10527_MOESM3_ESM.docx]

SI2-Fig-1: Phylogeny of Caleosin genes. CLO3 is marked as red, CLO1 as violet, CLO8 as green while CLO4, CLO6, CLO5 and CLO7 is present as a single cluster marked as blue.

SI2-Fig-2: Phylogeny of Steroleosin genes. HSD1 is given as red, HSD6 as violet, HSD5 as green, HSD2 as yellow and HSD4 as olive green.

SI2-Fig-3: Phylogeny of SEIPIN genes with SEIPIN-1 represented as green, SEIPIN-2 and 3 represented as violet and castor, olive and oil palm SEIPIN genes as green.


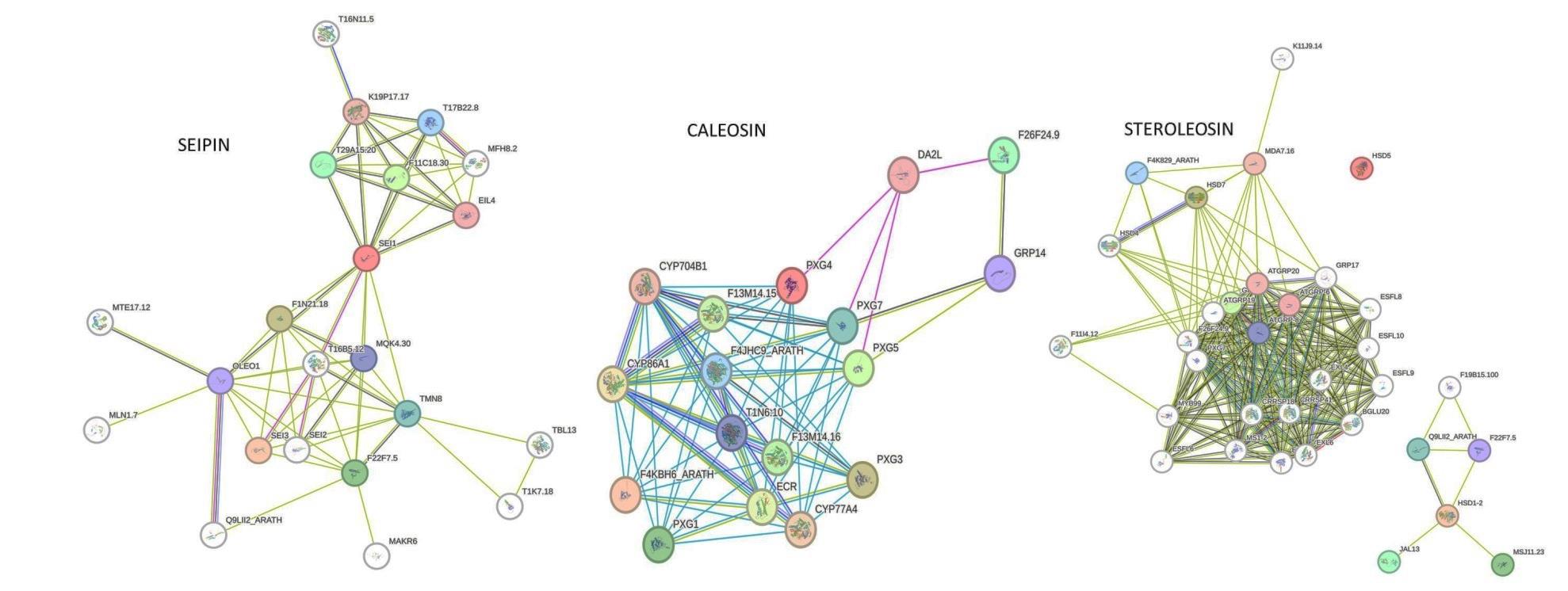


SI2-Fig-4: The protein-protein interaction of LD-associated steroleosins, caleosins and seipins and their close association with oleosins in LD organization and lipid metabolism.
