# Supplementary material for: Exploring selection signatures in the divergence and evolution of lipid droplet (LD) associated genes in major oilseed crops
Source: BMC Genomics. 2024 Jul 1;25:653. doi: 10.1186/s12864-024-10527-4 (PMC11218257; doi:10.1186/s12864-024-10527-4)
Supplement: Supplementary file 4 — Supplementary Material 4 [file 12864_2024_10527_MOESM4_ESM.docx]

SI-3 The MCMC phylogeny tree of LOX genes including all the ten species studied based on *A. thaliana* and *G. max c*lassification. Different subfamilies are given in separate colours; wherein 9S are represented as blue and 13S as red. Type II genes of 13S are represented as orange and Type II genes of 9S category is represented as brown.

**SI-1 The taxonomic classification of species studied.**

SI-9 The MCMC phylogeny tree of phospholipase D (PLD) genes including all the ten species studied based on *A. hypogaea, A. thaliana and O. sativa* classification. Different subfamilies are given in separate colours.

SI-10 The MCMC phylogeny tree of oleosin genes including all the ten species studied based on *A. thaliana, R. communis* and *G.hirsutum* classification. Different subfamilies are given in separate colours.

|  | TGL |
| --- | --- |
|  | SDP |

SI-11 The MCMC phylogeny tree of TAG lipase genes including all the ten species studied based on *A. thaliana* and *O. sativa* classification. Different subfamilies are given in separate colours
