# Supplementary material for: Exploring selection signatures in the divergence and evolution of lipid droplet (LD) associated genes in major oilseed crops
Source: BMC Genomics. 2024 Jul 1;25:653. doi: 10.1186/s12864-024-10527-4 (PMC11218257; doi:10.1186/s12864-024-10527-4)
Supplement: Supplementary file 5 — Supplementary Material 5 [file 12864_2024_10527_MOESM5_ESM.docx]

**Supplementary Information**

SI-1 The taxonomic classification of species studied.

SI-2. The protein characteristics including length of protein, molecular weight in KDa, iso-electric point (pI), GRAVY score and the location in the genome for lipoxygenases (A), phospholipase D (B), oleosin (C) and TAG-lipases (D).

SI-3 The MCMC phylogeny tree of LOX genes including all the ten species studied based on *A. thaliana* and *G. max* classification. Different subfamilies are given in separate colours; wherein 9S are represented as blue and 13S as red. Type II genes of 13S are represented as orange and Type II genes of 9S category is represented as brown.

SI-4 The subcellular localization of lipoxygenases (A), phospholipase D (B), oleosin (C) and TAG-lipases (D).

SI-5 The effect of natural selection as indicated by McDonald and Kreitman analysis (MK test). The parameters ω or Ka/Ks and the proportion of substitutions fixed by positive selection (α) were studied in lipoxygenases (A), phospholipase D (B), oleosin (C) and TAG-lipases (D). Significance test was done using Fisher's exact test and the P-values are given. Tajima’s D test conducted on each species based on gene families is given as 5E.

SI-6 GO enrichment analysis indicating biological process (BP), molecular function (MF), and cellular component (CC) classes as well as the KEGG pathways of of lipoxygenases (A), phospholipase D (B), oleosin (C) and TAG-lipases (D) in *A. thaliana* and *G. max*.

SI-7 The homologs identified in sesame variety improved Baizhima for Zhongzhi13 (refseq) gene sequences corresponding to lipoxygenases, phospholipase D, oleosin and TAG-lipases included in the study.

SI-8 The expression patterns of lipoxygenases (A), phospholipase D (B), oleosin (C) and TAG-lipases (D) in different tissues of *G.hirsutum*, *S. indicum*, *G. max* and *A. thaliana*. The final selection status of the individual genes are represented by colours where green represents ω>1 and α =positive (positive selection); blue represents ω<1 and α =positive and red represents ω<1 and α =negative (purifying selection).

SI-9 The MCMC phylogeny tree of phospholipase D (PLD) genes including all the ten species studied based on *A. hypogaea, A. thaliana and O. sativa* classification. Different subfamilies are given in separate colours.

SI-10 The MCMC phylogeny tree of oleosin genes including all the ten species studied based on *A. thaliana, R. communis and G.hirsutum* classification. Different subfamilies are given in separate colours.

SI-11 The MCMC phylogeny tree of TAG lipase genes including all the ten species studied based on *A. thaliana* and *O. sativa* classification. Different subfamilies are given in separate colours.

SI2-a : LD associated genes in oil palm.

SI2-b: Steroleosin genes identified in oil seed crops.

SI2-c: Caleosin genes identified in oil seed crops.

SI2-d: SEIPIN genes identified in oil seed crops.

SI2-e: The sub cellular localization of Caleosin, Steroleosin and SEIPIN proteins.

SI2-f The effect of natural selection as indicated by McDonald and Kreitman analysis (MK test). The parameters ω or Ka/Ks and the proportion of substitutions fixed by positive selection (α) were studied in lipoxygenases (A), phospholipase D (B), oleosin (C) and TAG-lipases (D) in oil palm as well as steroleosin, caleosin, and seipins in all the oil seed crops. Significance test was done using Fisher's exact test and the P-values are given.

SI2-g: The cis elements identified in the upstrem promoter region of Caleosin, Steroleosin and SEIPIN genes.

SI2-h: The expression patterns of Caleosin, Steroleosin and SEIPIN genes in different tissues.

SI2-i Significant GO terms associated with Caleosin, Steroleosin and SEIPIN genes

SI2-Fig-1: Phylogeny of Caleosin genes. CLO3 is marked as red, CLO1 as violet, CLO8 as green while CLO4, CLO6, CLO5 and CLO7 is present as a single cluster marked as blue.

SI2-Fig-2: Phylogeny of Steroleosin genes. HSD1 is given as red, HSD6 as violet, HSD5 as green, HSD2 as yellow and HSD4 as olive green.

SI2-Fig-3: Phylogeny of SEIPIN genes with SEIPIN-1 represented as green, SEIPIN-2 and 3 represented as violet and castor, olive and oil palm SEIPIN genes as green.

SI2-Fig-4 The protein-protein interaction of LD-associated steroleosins, caleosins and seipins and their close association with oleosins in LD organization and lipid metabolism.
